# Sexual Functioning and Sexual Health in Female Patients following Stroke: A Scoping Review with Implications for Rehabilitation

**DOI:** 10.3390/jpm14030267

**Published:** 2024-02-29

**Authors:** Desirèe Latella, Alessandro Grimaldi, Rocco Salvatore Calabrò

**Affiliations:** 1 IRCSS Centro Neurolesi Bonino Pulejo, S.S. 113 Via Palermo, C.da Casazza, 98124 Messina, Italy; desiree.latella@irccsme.it; 2 Department of Nervous System and Behavioural Sciences, Psychology Section, University of Pavia, Piazza Botta, 11, 27100 Pavia, Italy; alessandro.grimaldi01@universitadipavia.it

**Keywords:** sexual rehabilitation, stroke, female sexual function, quality of life

## Abstract

The impact of stroke on the sexual functioning of female survivors can arise from direct neurological effects or be triggered indirectly through various psychophysiological processes. Although an increasing number of publications exist, the topic of sexuality remains seldom discussed in the stroke literature, even though patients have expressed a need for sexual rehabilitation services. A literature review on sexual functioning in post-stroke females examining existing rehabilitation programs addressing patients’ sexuality and exploring the perspectives of nurses and caregivers on sexual issues constitutes a novel approach. Therefore, we conducted a scoping review to better investigate this hot topic. Studies were identified by searching Scopus, PubMed, Web of Science, Cochrane Library, PsychINFO, and Embase databases. Current data indicates substantial connections between stroke and female sexual dysfunction (SD), including factors like desire/libido, sexual satisfaction, and sexual intercourse. Some intervention programs have been created to provide specific guidance to healthcare professionals in addressing patients’ requirements for sexual recovery, although their adequacy remains uncertain. However, to date, there are neither specific rehabilitation programs for post-stroke female SD nor healthcare personnel trained to deal with post-stroke sexual issues adequately and efficiently. The incorporation of sexual rehabilitation into the overall rehabilitation process for stroke patients is crucial, ideally within an interdisciplinary framework. Despite being a fundamental aspect of post-stroke women’s lives, sexuality remains taboo.

## 1. Introduction

Stroke is a leading cause of increased mortality risk and disability worldwide [1]. It is responsible for 9–10% of all deaths [2,3,4,5]. Worldwide, it is estimated that 1/6 of people will experience a stroke in their lifetime [6,7,8,9]. Therefore, the burden of disease and the relational impact of disease upon stroke survivors and their caregivers are significant. Numerous studies have highlighted that individuals after a stroke (both ischemic and hemorrhagic strokes) encounter physical challenges (such as hemiparesis, hemi-hypoesthesia, dysphagia, sphincter incontinence, spasticity), cognitive–behavioral (aphasia, neglect, apraxia, depression, etc.), emotional and relational consequences [10,11,12]. Problems with sexual health and function are also frequent, and they can be related to the direct effect of the lesion (e.g., loss of desire following frontal lobe stroke), or secondary to either the physical impairment or the patients’ and/or partners’ reaction to the disease [13,14,15].

Sexual activity is important to health and well-being and correlates with greater life enjoyment for older adults. Although the frequency of sexual activity tends to decline with age as a consequence of physiological changes, older adults may still be sexually active. The presence of pre-stroke comorbidities is a critical factor to consider when evaluating the impact of stroke on sexual function. Common medical conditions among older women, including asthma, chronic obstructive pulmonary disease, heart disease, diabetes mellitus, depression, arthritis, and cancer, can impact sexual experiences. Similarly, medications commonly prescribed to the elderly, such as antidepressants and antiepileptics, may diminish sexual function. These conditions and medication use can predispose individuals to sexual difficulties, which may be exacerbated following stroke. Understanding how pre-stroke comorbidities influence sexual function is crucial for delivering comprehensive and tailored care to stroke patients [16,17,18,19,20,21,22,23,24].

The growing literature underlines an association between decreased sexual activity following stroke and the level of disability following stroke. Sjogren’s study highlighted that the level of independence in activities of daily living serves as a reliable predictor of sexual activity [25]. These findings were corroborated by Kimura and Murata’s research, which identified a positive correlation between functional disabilities and sexual dysfunction (SD). In a survey involving post-stroke patients [26], 55% of spouses identified hemiparesis as the primary obstacle to sexual activity, while 29% cited spasticity, 19% reported sensory deficits, and 14% mentioned aphasia as the main reasons for discontinuing sexual activity. In Cheung’s study involving post-stroke patients with mild to no disability, it was observed that over 50% of women reported experiencing SD before the stroke [27]. However, following stroke, the prevalence of SD significantly rose to 75%. Before the stroke, one-third of the women reported difficulty in achieving orgasms, whereas after the stroke, 50% of the women reported orgasmic difficulties.

Various factors across a wide spectrum contribute to sexual functioning and stroke itself was identified as a factor associated with a decrease in sexual functioning, independent of gender [28,29,30]. Lesion locations, severe hemiplegia and hypoesthesia, aphasia, spasticity, and sphincteric incontinence are the main causes of stroke-related SD. For example, a significant correlation was found between SD and left or right hemisphere lesions. Rates of SD increased among stroke patients dealing with other clinical issues. Furthermore, the use of medications to manage post-stroke comorbidities like depression, hypertension, and other heart problems also contributed to SD [30,31,32]. Nonetheless, the literature results are inconsistent and controversial, and it can be concluded that SD must be attributed to multifactorial etiology, also including the psychosocial and relational context [33]. Indeed, coping with stroke-related disability in the community is not only important to the patients themselves but also to their partners/caregivers.

Although sexuality plays a crucial role in an individual’s life, encompassing various aspects like emotions, partner relationships, and self-acceptance, few studies in the literature focused on these fundamental aspects in female patients with stroke [34]. Moreover, health professionals do not consider sexual functioning as an integral part of the rehabilitation process, although sexual wellness contributes to the well-being and quality of life of both patients and their spouses [35,36,37]. Then, knowledge of human sexuality, as well as myths and beliefs surrounding patients with a disability, is fundamental for any healthcare professionals working in the rehabilitation field [38]. Finally, the treatment of SD in female patients with stroke is another important issue to define. Sexual rehabilitation following stroke encompasses the evaluation of prevailing sexual challenges, offering guidance on related concerns, and facilitating the resumption of sexual activity. This kind of rehabilitation is individualized and should employ a holistic approach, including psychology, physiotherapy, occupational therapy, social work, and nursing, as well as applying a biopsychosocial model [39,40].

In this scoping review, we assessed the literature regarding sexual functioning in female patients following stroke. We investigated risk factors that contribute to SD, its impact on the satisfaction and quality of life, rehabilitative programs, and the approach of caregivers/health professionals to stroke survivors.

## 2. Materials and Methods

### Search Strategy

**Figure 1:**
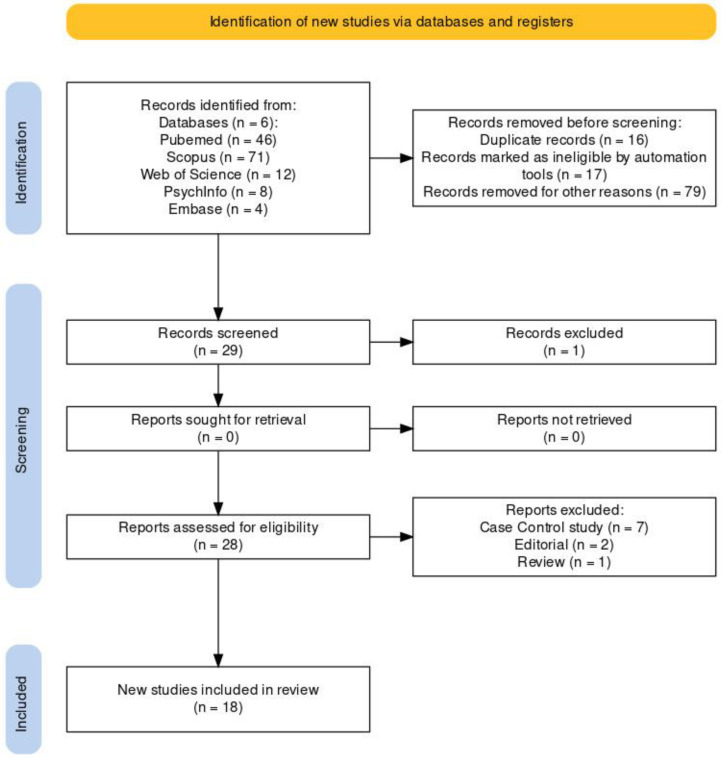
PRISMA 2020 flow diagram for updated systematic reviews, which included searches of databases and registers only.

The studies were identified by searching Scopus, PubMed, Web of Science, Cochrane Library, PsychINFO, and Embase databases. The inclusion criteria were (i) female (18 years and older) patients affected by any kind of stroke (including both ischemic/hemorrhagic stroke and TIAs; recurrent and accidental*)*; (ii) randomized clinical trials (RCT) and pilot studies; (iii) written in the English language; and (iv) published in a peer-reviewed journal. Exclusion criteria were (i) case reports, editorials, and narrative and systematic reviews; (ii) studies involving men or dealing with gender identity; and (iii) papers dealing with psychiatric patients. All the studies fulfilling our selected criteria and published between 2013 and 2023 were evaluated for possible inclusion. The search combined the following terms: “sexual behavior” AND “female” AND/OR “stroke rehabilitation”, AND/OR “female stroke patients” AND “sexual dysfunction” AND/OR “sexuality”. The search terms were identified as title and abstract. After duplicates had been removed, all articles were evaluated based on title and abstract (See Figure 1). Papers were screened by two authors (LD and AG). In the case of disagreement, the concern was solved by a third reviewer (RSC).

This review was registered with a DOI (https://doi.org/10.17605/OSF.IO/KPZ7C) (accessed on 6 November 2023) on the Open Science Framework (OSF).

## 3. Results

### 3.1. Assess Quality of Included Studies—Risk of Bias

The risk of bias in controlled studies was assessed using a revised Cochrane risk of bias (RoB 2) (2019) tool [41], which comprises five domains: (i) bias arising from the randomization process; (ii) bias due to deviations from the intended intervention; (iii) bias due to missing outcome data; (iv) bias in the measurement of the outcome; and (v) bias in the selection of the reported result. Further, the risk of bias in non-randomized studies of exposures (ROBINS-E) (2023) tool [42] comprises seven domains: (i) bias due to confounding; (ii) bias arising from measurement of the exposure; (iii) bias in selection of participants into the study (or into the analysis); (iv) bias due to post-exposure interventions; (v) bias due to missing data; (vi) bias arising from measurement of the outcome; and (vii) bias in the selection of the reported result. We identified only 2 studies with a low risk of bias and robust methodologies, while 16 studies presented a high risk of bias and weak methodologies.

After the first screening based on the title and abstract, we removed one study because the manuscript did not divide the results for men and women by gender, while the title seemed to concern female issues. Finally, we found 18 studies dealing with sexual functioning following stroke in female patients, of which 12 studies focused on SD following stroke, 2 studies focused on sexual rehabilitation programs following a stroke event, and 4 studies dealt with health professionals’/spouses’ perspectives on sexual female functioning post-stroke during rehabilitation programs.

### 3.2. Female Sexual Functioning following Stroke

Stroke has been associated with a considerable decrease in overall sexual satisfaction among women, as indicated by Azanmasso et al. The research suggests that the risk SD increases by 15 times after a stroke [43]. The prevalent forms of SD following stroke include orgasmic dysfunction (up to 75% of cases), difficulties with lubrication (50–77% of cases), substantial issues with desire or libido, and a decreased frequency of sexual activity [44].

A biopsychosocial approach provides a comprehensive understanding of post-stroke SD. Beyond the direct neurological impact of a stroke on the neurophysiology of the sexual response, there are additional factors associated with strokes that can indirectly affect sexual activity. These factors include physical limitations resulting from neurological lesions [44].

Lever and Pryor have described considerations of the self and their own body, physical limitations during sexual acts, and sexual activities with or without genital involvement as limitations on female sexuality [44]. The patients referred to their upper and lower limbs as dead/deformed/alien with details outlining alterations in tone, sensation, microcirculation, spasticity, and movement limitations. Other physical alterations included facial paralysis, dysphagia, mouth dripping, urinary incontinence, altered cognitive status, and aphasia [44]. Fatigue was a common physical problem in women during post-stroke sexual experiences. For many women, feeling older, the fear of having another stroke during sex, and communicating to their partner that they are a stroke survivor contributes to the impact on female sexuality limiting possibilities for expressing their sexuality and their sense of womanhood influencing overall wellness [45,46]. After a stroke, many women have reported a tendency to neglect their personal hygiene and physical appearance, resulting in a notable impact on their intimate relationships. The occurrence of a stroke alters a woman’s appearance, sensuality, and overall sense of femininity. Kvigne and Kirkevold delved into the experiences of female stroke survivors with their lived bodies, revealing a thematic classification of their bodies as unpredictable (manifesting as nonspontaneous, vulnerable, defenseless, unreliable, and betraying) and demanding (requiring significant time, imposing limitations, fostering dependence, and drawing attention). These changes also prompted the perception of the body as extended, necessitating the incorporation of various aids and helpers into the women’s lives. Self-devaluations of the body were common because of low self-esteem and wrong beliefs about one’s physical appearance [47,48]. In the immediate post-stroke period, women suffered greatly from their physical condition, so they committed themselves to taking care of their bodies to be more attractive. The need to feel desirable, attractive, and pretty was an urge shared by post-stroke women. These needs were influenced by self-perception and judgments of self [49,50,51]. In a survey, some women reported that the sexual response and sexual desire, arousal, and orgasm remained unchanged following stroke. Others reported a slowing down or lessening of intensity relative to desire, arousal, and orgasm [51]. Depression, anxiety, and emotional lability contribute to the etiology of post-stroke SD. A large proportion of patients recovering from a stroke report worse well-being. As evidenced by the research review, these psychological factors can have an impact on patients’ sexual functioning, sexual desire, and satisfaction [52].

Sjogren and Fugl-Meyer conducted an assessment involving 110 post-stroke individuals to examine alterations in their sexual activities. The findings revealed that approximately 68% of patients noted a reduction in sexual activity, with 32% indicating a complete cessation of such activities [34]. In another study, Sjogren et al. highlighted that women reported a significant decrease in the frequency of intercourse, as well as reduced durations of both foreplay and intercourse. Additionally, 75% of the female participants reported a decline in orgasmic function and a decrease in sexual drive [25]. Recent cross-sectional studies conducted in the United States, Italy, Korea, and Turkey explored the impact of stroke on sexual activity. These studies indicated that 14–50% of patients abstained from sexual activity for 3 months post-stroke. Among those who resumed sexual activity, a restart typically occurred 3–6 months after the stroke event [53].

However, in most patients, despite the unchanged sexual desire and the importance of sexual activity, physical alterations following stroke limited how women could participate in sexual activity with the involvement of genitals. Physical limitations impeded penetration and active participation in sexual acts. Even if the female had mobility, the options for sexual intercourse were constrained in terms of positions. Female sexuality extends beyond intercourse to encompass various intimate activities like touching, massaging, cuddling, holding hands, snuggling, skin-to-skin contact, and kissing. These intimate endeavors held significant value and were deemed crucial by women in the aftermath of a stroke. These activities were desired by women in meaningful intimate relationships [54,55]. The importance of lovingness and tenderness in the context of a stable relationship was the prerogative to achieve enjoyment. Also, altered sensations impacted the women’s experience of being touched, massaged, cuddled, and kissed. For example, hemiparesis limited the experience of touching, holding hands, or cuddling. These intimate activities along with sexual acts with genital involvement indicate that women considered sexuality on a broad spectrum, from sexual desire and activity to decreased sexual satisfaction. Through assessing 192 post-stroke patients and 94 spouses, a study revealed a significant decline in satisfaction from the pre-stroke rate of 90% to less than 50% [56].

In the extended aftermath of a stroke, female survivors encounter heightened obstacles in motor tasks, participating in events, and actively engaging in social interactions. This gender gap appears to stem from the lack of social assistance, a crucial factor known to enhance recovery after a stroke. With women potentially facing greater challenges in obtaining assistance for daily activities, they are more susceptible to social isolation, which in turn carries adverse health ramifications [26].

Another contributing factor to sex differences in stroke outcomes is the presence of depression, which has been linked to increased post-stroke disability. Research indicates that following stroke, individuals with depression tend to perceive lower levels of social support, subsequently affecting their quality of life and sexual health [57]. The observed sex differences in stroke-related outcomes suggest that women may experience more severe functional limitations due to social isolation and depression [58].

**Figure 2:**
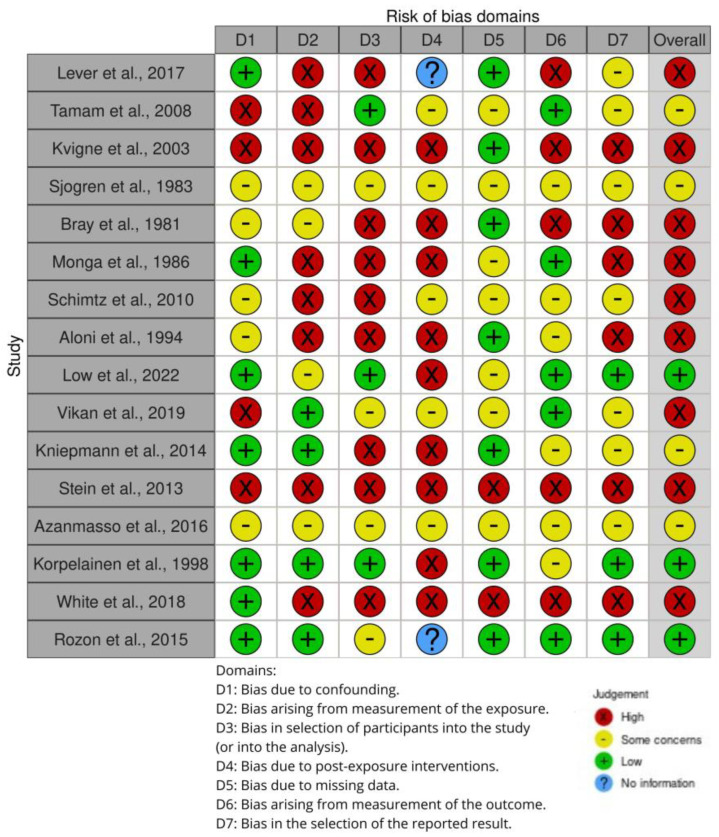
Shows the risk of bias (ROBINS-E) of studies regarding the changes in sexual functioning and SD experienced by post-stroke women [34,43,44,45,46,47,48,51,54,55,57,58,59,60,61,62].

In addition, we evaluated the quality of the included studies (Table 1), as shown in Figure 2 for observational studies. We found that the risk of bias was present in all seven domains. These studies [34,43,44,45,46,47,48,51,54,55,57,58] did not adequately manage the confounding factors, and there were biases arising from the measurement of the exposure, in the selection of participants for the study or the analysis, due to post-exposure interventions and missing data, from the measurement of the outcome and in the selection of reported result procedures.

### 3.3. Rehabilitation

The defined objectives of sexual rehabilitation following stroke encompass the evaluation of prevailing sexual challenges, offering guidance on related concerns, and facilitating the resumption of sexual activity post-stroke [63,64]. This rehabilitation is individualized, employing a holistic approach that integrates psychology, physiotherapy, occupational therapy, social work, and nursing. It adheres to a person-centered, time-based, and functionally oriented design, aiming to enhance activity and participation (social integration) through the application of a biopsychosocial model. Counseling constitutes a significant component of sexual rehabilitation, addressing concerns related to sexual performance, the impact of medication and comorbid conditions on sexual function, and specific psychological or interpersonal factors [65]. This counseling can take place individually or in a group setting. Beyond counseling, sexual rehabilitation may incorporate various elements of physical rehabilitation. For instance, a physiotherapist may provide mobility training to enhance bed mobility for optimal sexual positioning and facilitate bed transfers. Spasticity management, such as using a bolster, may also be part of the rehabilitation process. Physiotherapy also offers practical guidance, including advice on optimal timing for sexual activity (such as in the morning when fatigue is minimized), management of bladder and bowel issues, and addressing fatigue through physical support with pillows.

Sexual rehabilitation is typically administered by appropriately trained healthcare professionals within a multidisciplinary team, involving the stroke survivor either alone or with their partner. The delivery of sexual rehabilitation can take various formats, encompassing oral information, visual aids, written materials, and audio-visual and hands-on training [66]. Sexual rehabilitation may also be provided using cognitive-behavioral therapy targeting psychological and physical aspects of sex and intimacy [67], given that most of the pharmacological treatments have focused on males. Moreover, few studies have taken into consideration the patient’s partner, even if a relationship can be affected by catastrophic events like stroke, and therefore couples counseling is fundamental.

Counseling and psychotherapy play a role in alleviating anxiety and fear associated with sexual problems, offering reassurance regarding concerns that engaging in sexual activity might trigger another stroke. This can contribute to enhanced confidence in one’s sexual abilities. Complementary medicine interventions are another avenue, potentially increasing nitric oxide levels and consequently improving sexual function [68]. To date, international guidelines recommend the assessment of sexual function and its management following stroke [69,70,71]. Considering sexuality as an integral part of rehabilitation and implementing standard care procedures could be important to encourage employees to address any doubts and fears about the sexual rehabilitation of patients and their partners. The use of models is very important to evaluate and address the sexual health of individuals. The PLISSIT model consists of four steps for addressing sexual concerns: Permission, Limited Information, Specific Suggestions, and Intensive Therapy. It provides sexual counseling to healthcare professionals through its ease of use and systematic approach. Annon’s model, developed in 1976 as a guide to the resolution of sexual problems, was revised by Taylor and Davis in 2006. The model consists of four phases. The first phase is Permission. At this stage, patients are encouraged to express their concerns about sexuality and sexual health. The second phase is “Limited Information”, which involves an analysis of ideas about sexuality, the effects of the disease, and treatments on sexual function. It provides accurate information to address their concerns about sexuality more effectively. The “Specific Suggestions” phase includes providing individuals/spouses with specific information and recommendations for a more fulfilling sex life. It is particularly effective against arousal and orgasm disorders and dyspareunia. The recommendations for different positions, interventions, and lubricants can be useful. The “Intensive Care” phase is the final one. If the first three phases of the model are unsuccessful, the intensive care phase is the solution [72]. In a study, Ng et al. used the PLISSIT program in the treatment of SD in post-stroke subjects. Programs were individually tailored. The content was similar to the above-mentioned sexual rehabilitation programs and included an information phase regarding common changes in sexuality post-stroke counseling on fears and concerns regarding post-stroke sexuality; questioning conventional perspectives on sexuality and sexual achievement; providing advice and techniques to reduce post-stroke SD, including selecting appropriate timing, the side effects of medications on sexual function, identifying safe and comfortable sexual positions, decreased vaginal lubrication, urinary continence concerns, or erectile challenges [73].

**Figure 3:**
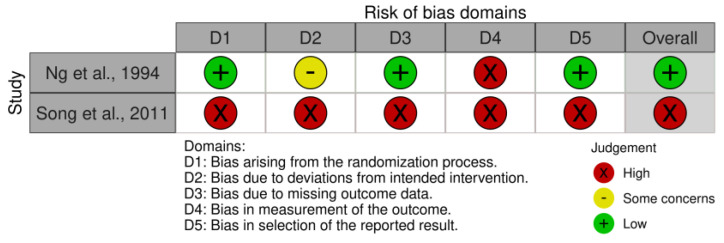
Shows the risk of bias (RoB 2) of studies regarding the sexual rehabilitation programs for women following stroke [67,73].

In addition, we evaluated the quality of the included studies (Table 2), as shown in Figure 3 for RCT studies. We found that the risk of bias was present in all five domains. These studies [67,68,69,70,71,72,73] did not adequately manage the randomization procedures, and there were biases due to deviations from the intended intervention, missing outcome data, in the measurement of the outcome, and the selection of reported result procedures.

### 3.4. Health Professional and Spouses’ Perspectives

Growing evidence in the literature points to the barriers that hinder rehabilitation personnel from addressing issues related to sexuality in post-stroke patients. The lack of ad hoc training, the reticence to address the topic of sexuality, the lack of standardized procedures, the timing of intervention, and specialized skills in sexual rehabilitation are the main obstacles to addressing sexual concerns and effective sexual rehabilitation. Schmitz and Finkelstein identified seven distinct themes concerning perspectives on sexual issues following stroke [59].

The primary obstacles to rehabilitation encompass alterations in functional and relational aspects of sexual functioning, the lack of discourse or challenges in addressing sexual topics by rehabilitation professionals, the absence of sexual policies in managing post-stroke sexual functioning, and the failure to recognize the significance of sexual issues [59]. A survey by Vikan et al. evidenced that the absence of a sexual health policy serves as a primary obstacle to incorporating evidence-based practices into stroke rehabilitation programs. In fact, this multicenter study indicates that in centers with sexual health policy, health professionals and administrative leaders are more trained and careful concerning sexuality post-stroke. Therefore, this information indicates that the absence of sexual health policies leaves healthcare professionals in a state of uncertainty regarding their duties, lacking guidance, and facing undefined roles and responsibilities in clinical practice. This condition permits the sharing of responsibility within the rehabilitation team. Therefore, an interdisciplinary approach is essential for addressing sexuality after a stroke. The lack of sexual policies in healthcare services is not a rule, but ranges widely. That healthcare services do not follow recommendations on implementing sexual health, where they are present, in standard care is concerning [60]. In addition, there are misconceptions about sexuality by health professionals and patients who fear being judged upon sharing their concerns, schedule conflicts, time restrictions, and the weight of other professional obligations that contribute to complicating the issue. Vikan et al. highlight that the majority of health professionals report having little or no knowledge about sexual health following stroke [60]. Physical limitations arising from stroke, such as aphasia, may contribute to barriers that hinder patients from expressing their concerns or posing questions about post-stroke sexuality. The literature reviewed indicates that stroke survivors would attend information sessions about the implications of post-stroke sexuality, as well as to understand how medication may affect their sexual function, and grasp the impact of potential comorbidities such as diabetes and hypertension on their sexual function following stroke. Furthermore, the partners of stroke survivors frequently seek answers to questions about engaging in sexual activity with their affected partner or look to develop new coping strategies. In certain situations, partners find themselves feeling isolated as they undertake the role of caregiver during the stroke recovery process [73,74]. In a study examining post-stroke changes in sexuality, Kniepmann et al. identified certain concerns from the perspective of spouses/partners. These concerns revolved around maintaining closeness and togetherness, as well as redefining sexuality and intimacy after a stroke, the presence of resources or support about sexuality, and the lack of trained health professionals on the issue. Kniepmann confirmed prior results, revealing that many relationships faced challenges post-stroke related to difficulties in communication and relationship changes [61]. Spouses of stroke survivors redefined the intimate relationship, preferring holding, gazing into one another’s eyes, or lying together rather than engaging in physical sex. However, intercourse is not the only important part of a relationship, and it was replaced with increased intimacy following stroke. The couples could be encouraged to explore other dimensions of intimacy. The discharge information given to stroke survivors and their partners lacks sufficient attention to these aspects of sexuality and intimacy.

Training a team on the topic of post-stroke sexuality is necessary. Kniepmann’s study indicates patients want to feel free to talk about post-stroke sexuality and be confident that they are talking to trained professionals. This underscores findings from prior research, indicating that 50–75% of stroke survivors and their spouses expressed an interest in receiving sexual counseling during rehabilitation programs, yet only 15.2% received such services [61,62]. Various models, including the PLISSIT model [72], the Sexual Health Model [75], and the extended PLISSIT model [76], offer introductory training on addressing sexuality in diverse healthcare settings.

Utilizing such models in hospitals and rehabilitation settings could benefit clinicians in addressing concerns about intimacy and sexuality post-stroke. Educating healthcare professionals means that they will be more prone to discuss sexuality and intimacy with their patients [62,75]. Moreover, the nurses/health professionals are more predisposed to care for physical functioning, to help the patient recover normal functions and learn skills to do everyday tasks, manage self-care, and obtain independence [77,78]. However, addressing SD is an important part of stroke rehabilitation and most patients wish to receive information about sexuality. Finally, gender differences were paid little attention by nursing [48]. These aspects must be considered in developing guidelines and for providing care for sexual rehabilitation. Considering sexuality as an integral part of rehabilitation, implementing standard care procedures could be important to encourage employees to address any doubts and fears about the sexual rehabilitation of patients and their partners.

In addition, we evaluated the quality of the included studies (Table 3), as shown in Figure 2 for observational studies. We found that the risk of bias was present in all seven domains. These studies [59,60,61,62] did not adequately manage the confounding factors, and there were biases arising from the measurement of the exposure, in the selection of participants for the study or the analysis, due to post-exposure interventions and missing data, from the measurement of the outcome and in the selection of reported result procedures.

Figure 3 shows the risk of bias (RoB 2) of studies regarding the perspective of healthcare professionals and partners toward post-stroke sexual issues.

## 4. Discussion

As far as we know, this is the first scoping review on the implications that sexual functioning and health may have in female patients with stroke. All reviewed studies underline the role of psychological, social, personal, cultural, and biological factors in female sexual function. The primary hindrances to sexual activity can be categorized as direct and indirect. Direct obstacles involve physical issues like diminished stamina, mobility constraints, reduced sensitivity, and general pain. Indirect obstacles pertain to various stages of the sexual response cycle, encompassing arousal, plateau, orgasm, and resolution. Then, to understand changes in sexual functioning in post-stroke women, it is necessary to consider both sexual anatomy and the various stages of sexual activity [79]. The sexual response cycle may not be as linear in women as in men [80]. A group of researchers denied the applicability of the linear model to real-life sexual experiences because human sexuality is not strictly dependent on physical factors [81,82,83,84]. In 2000, Basson proposed a model of female sexual response, defined as cyclical. The main novelty was that, in women, erotic desire or fantasy does not always represent the start of sexual activity, but in the event of sensations of emotional intimacy, the woman seeks sexual stimulation. This is a responsive desire, as opposed to a spontaneous one. Basson’s model underlines that desire and arousal overlap, considering that for many women the two aspects are difficult to distinguish [85,86]. Positive sexual experiences ease the sexual response cycle and allow one to transition from a state of sexual neutrality into sexual desire, arousal, and satisfaction [80]. Some conditions, such as the ictus itself and drug therapies, can influence the stages of the sexual cycle and interfere with sexual pleasure and satisfaction or may lead to painful experiences. According to some authors, sexual arousal is a cognitive elaboration of a stimulus experienced as pleasant with peripheral, genital, and extra-genital implications [59]; therefore, diseases such as stroke can impair the functioning of nerve pathways, making it difficult for the sexual cycle to begin or progress. Following a stroke event, it is important to reframe the concept of intimacy for patients, emphasizing touch, pleasure, and emotional connection; on the other hand, it is important to moderate the traditional focus on sexual functioning [87]. A broader perspective on intimacy can help individuals and their partners maintain a satisfying and fulfilling connection despite physical limitations or changes resulting from stroke. The rehabilitation team, including physiatrists, psychologists, and speech, occupational, and physical therapists, can suggest changes related to the type of sexual activity and frequency as they do for other activities of daily living, using the PLISSIT model [72]. PLISSIT encourages healthcare professionals to offer precise information and recommendations regarding sexual concerns. The rehabilitation team should be equipped to address patients’ inquiries about their current sexual functioning and expectations, along with those of their partners or spouses. Schimtz’s study emphasizes the existence of unmet rehabilitation needs for both stroke survivors and their spouses or partners regarding sexual problems and concerns post-stroke [59]. This is underscored by most participants reporting a lack of opportunity to discuss sexuality with anyone during the rehabilitation process. The authors also highlight the lack of adequate preparation on post-stroke sexual changes and reticence to address this topic, hindering the efficacy of sexual rehabilitation programs. Rehabilitation specialists must learn to recognize and question patients regarding the domains of sexual cycle/response and to provide concrete referrals to sex therapy. Physical therapists trained in pelvic floor rehabilitation should be included on the rehabilitation team to assist patients who report pelvic or genital pain with sexual intercourse. Within the team, the psychologist should address the psychosocial barriers experienced by patients and partners/spouses [59]. The significant changes in family and social roles, including the loss of role identity by the patient and the new caregiving role of the partner, should be acknowledged. Furthermore, healthcare professionals must recognize and help patients to manage common sexual concerns including performance anxiety, fear of another stroke, and depression. Despite it being easy to access knowledge on SD, in general, healthcare professionals remain the most authoritative point of reference for patients. Nevertheless, the information regarding sexual function post-stroke, potential solutions, and reassurance is typically broad, lacking specificity, and frequently lacking evidence-based support. Many patients tend to undergo the rehabilitation process passively.

According to our experience in the field of rehabilitation and considering the treatment of sexual changes in post-stroke women, we believe it is useful to integrate the clinical practice addressing sexuality post-stroke change either during and/or in the post-hospitalization rehabilitation period, but a standardized questionnaire is needed to better investigate female SD and relationships problems following stroke.

Then, healthcare professionals working in the rehabilitation field must receive adequate training about sexual behavior and sexual health to overcome false myths and personal beliefs about sexuality and disability. Collaboration of rehabilitation professionals with sexual medicine specialists including sexologists and sex therapists may assist in addressing these sexual concerns in post-stroke rehabilitation. Couples sexual counseling and pelvic floor muscle training should enter the clinical practice when dealing with female patients affected by post-stroke SD [88].

In addition to sex education with specific training for both patients and their caregivers, people with stroke may benefit from a “sex coach”, i.e., a trained professional who helps people with sexual, intimacy, and relationship issues, including a sexless marriage, low libido, and SD. In fact, for anyone who has experienced changes resulting from a disability and does not have a partner to work with, the coach or surrogate can help explore and develop sexual potential in disabled people, like those affected by stroke. Finally, soon, following the growing field of robotics and AI, social and sex robots may be an alternative solution for overcoming the mental and social taboos related to this “hot” topic.

## 5. Conclusions

Female SD after a stroke can be a profound challenge for survivors and their partners. Beyond the physical implications, it can affect self-esteem, relationships, and overall quality of life. The emotional toll of grappling with changes in intimacy adds another layer of complexity to the recovery process. Understanding and addressing these aspects are crucial for a more comprehensive and supportive approach to post-stroke care. Addressing sexual issues in female stroke patients is essential for a holistic approach to their well-being. This is not just about physical recovery, but also about preserving the emotional and social aspects of their lives. Our scoping review highlights that it is necessary to increase knowledge about the management of sexual issues in female patients following stroke, as these issues constitute a crucial aspect of the patient’s relationship and social and emotional well-being.

## Figures and Tables

**Table 1:** Main studies on the changes in sexual functioning and SD experienced by post-stroke women.

Study	Design	Location	Patients	Mean Age	Major Findings
Lever et al., 2017 [44]	Descriptive qualitative study	Australia	9 female post-stroke survivors	31 to 70 years	The women experienced bodily alterations: an assault on the female sense of self and limitations on possibilities for enacting female sexuality.
Azanmasso et al., 2016 [43]	Cross-sectional study	Nigeria	67 stroke patients 67 healthy controls	54.97	Women who have experienced a stroke may experience various forms of sexual problems, including a lack of sexual desire, trouble becoming aroused, vaginal dryness, difficulty reaching orgasm, and pain during intercourse. Several factors can influence sexual functioning after a stroke, including the extent of the stroke’s damage, ongoing high blood pressure, use of blood pressure and antiplatelet medications, anxiety levels, occupation, and marital status.
Tamam et al., 2008 [45]	Descriptive qualitative study	Turkey	103 stroke patients	57.9	Post-stroke vaginal lubrication, orgasm frequency, and overall satisfaction were all statistically decreased in female stroke survivors.
Stein et al., 2013 [48]	Cross-sectional study	Stroke Rehabilitation Research Registry	38 stroke survivors (23 male and 15 female)	55.1	58% of respondents indicated that their sexual function had declined post-stroke.
Aloni et al., 1994 [46]	Randomized control trial	Israel	13 post-stroke females	45.5	In this research, the majority of women‘s post-stroke experience challenges related to feeling older, concerns about the risk of another stroke during sexual activity, and difficulties in communicating their survivorship to their partners. These factors collectively contribute to restricting opportunities for expressing their sexuality and shaping their sense of womanhood, ultimately impacting their overall well-being.
Kvigne et al., 2003 [47]	Descriptive study	Norway	25 post-stroke women	37 to 78 years	Female stroke survivors encountered profound, unsettling, and somewhat incomprehensible transformations in their bodies both at the onset of the stroke and throughout the recovery process.
Sjogren et al., 1983 [34]	Descriptive study	Sweden	36 female stroke	53.8	Women experienced a significant decrease in the frequency of intercourse, as well as reduced durations of both foreplay and intercourse. Additionally, 75% of the female participants reported a decline in orgasmic function and a decrease in sexual drive.
Korpelainen et al., 1998 [51]	Prospective study	Finland	50 stroke patients (38 men, 12 women	53.5	The presence of a stroke commonly led to a decrease in libido and coital frequency, as well as challenges with erection, ejaculation, vaginal lubrication, orgasm, and overall satisfaction with sexual life for both of the individuals affected by the stroke.
Bray et al., 1981 [54]	Descriptive study	Not mentioned	35 patients (24 men and 11 women)	-	Most stroke survivors experience sexual dysfunction following stroke.
Monga et al., 1986 [55]	Qualitative study	Not mentioned	113 patients (78 men, 35 women)	68.0	The primary factor frequently identified as contributing to a decrease in sexual activity was the concern that engaging in sexual intercourse could negatively impact blood pressure and potentially trigger another stroke
Rozon et al., 2015 [57]	Longitudinal study	Not mentioned	81 post-stroke female	63.3	This study examines changes in sleep, driving, employment, relationships, and leisure in the first year after a mild stroke. It explores the association between the presence of depressive symptoms and improvement in participation 6 months later. The results showed that all of the life habits are improved at 6 months and 1 year, except for having a sexual relationship.
White et al., 2018 [58]	Retrospective study	USA	641 women stroke survivors	66.90	The functional outcomes of women stroke survivors were significantly worse than men. The specific factors that contribute to sex differences in stroke-related outcomes are not entirely clear.

**Table 2:** Main studies on sexual rehabilitation programs for women following stroke.

Study	Design	Location	Patients	Mean Age	Major Findings
Song et al., 2011 [67]	Clinical trial study	South Korea	46 couples	40–46 years	The sexual rehabilitation intervention program led to improvements in sexual knowledge, sexual satisfaction, frequency of sexual activity, cognitive function, and performance in daily living activities. This indicates a significant enhancement in both sexual satisfaction and the frequency of sexual activity as a result of the devised program.
Ng et al., 2017 [73]	Randomized controlled trial	Australia	68 participants	62	The provision of written information alone appears to be as effective as a 30 min individualized sexual rehabilitation program in an inpatient setting. A total of 51% of the participants wished to receive information about sexual changes following stroke.

**Table 3:** Main studies on the perspective of healthcare professionals and partners towards post-stroke sexual issues.

Study	Design	Location	Patients	Mean Age	Major Findings
Schmitz et al., 2010 [59]	Descriptive study	USA	15 stroke survivors 14 partners of stroke survivors	65 years	This study identified two main clusters of the effects of stroke on sexual life, namely physical/functional changes and relationship changes. Various challenges were associated with addressing sexual issues during rehabilitation, including the reluctance of patients and providers to discuss sexual matters, limited discourse on post-stroke sexuality, the necessity to customize education to the specific needs of individuals or couples, the importance of provider rapport and competence, and the timing of post-stroke sexual education.
Kniepmann et al., 2014 [61]	Cross-sectional study	Cognitive Rehabilitation Research Group database	20 caregivers	-	This study suggests that being involved socially is important for health promotion for caregivers and enhances the health of the stroke survivor and the entire family.
Vikan et al., 2019 [60]	Cross-sectional study	Palestine, Israel, USA, Russia, China, Sweden	323 health professionals	<30 years (30%) 30–49 years (52%) ≥50 years (18%)	Healthcare personnel employed at facilities with elevated scores in sexual health policy within stroke rehabilitation exhibited increased knowledge and comfort in addressing sexual health matters. They also expressed a more favorable view of workplace sexual health policies compared to personnel at centers with lower scores in these aspects.
Low et al., 2022 [62]	Cross-sectional study	United States, Canada, Australia, New Zealand, United Kingdom, Ireland, Singapore, South Africa	958 stroke rehabilitation professionals	40.7	This study emphasizes the neglect of sexuality in stroke rehabilitation, underscoring the necessity for a thoughtful approach to the timing and content of educational interventions.

## Data Availability

Not applicable.
